# Modeling Provincial Covid-19 Epidemic Data Using an Adjusted Time-Dependent SIRD Model

**DOI:** 10.3390/ijerph18126563

**Published:** 2021-06-18

**Authors:** Luisa Ferrari, Giuseppe Gerardi, Giancarlo Manzi, Alessandra Micheletti, Federica Nicolussi, Elia Biganzoli, Silvia Salini

**Affiliations:** 1 Department of Statistical Science, University College London, London WC1E 6BT, UK; luisa.ferrari.20@ucl.ac.uk; 2 Department of Economics, Management and Quantitative Methods, University of Milan, 20122 Milan, Italy; giuseppe.gerardi@unimi.it; 3 Department of Economics, Management and Quantitative Methods and Data Science Research Center, University of Milan, 20122 Milan, Italy; federica.nicolussi@unimi.it (F.N.); silvia.salini@unimi.it (S.S.); 4 Department of Environmental Science and Policy and Data Science Research Center, University of Milan, 20122 Milan, Italy; alessandra.micheletti@unimi.it; 5 Department of Clinical Sciences and Community Health and Data Science Research Center, University of Milan, 20122 Milan, Italy; elia.biganzoli@unimi.it

**Keywords:** COVID-19, SIRD-derived models, Italy, EU NUTS-3 regions, epidemic data

## Abstract

In this paper, we develop a forecasting model for the spread of COVID-19 infection at a provincial (i.e., EU NUTS-3) level in Italy by using official data from the Italian Ministry of Health integrated with data extracted from daily official press conferences of regional authorities and local newspaper websites. This data integration is needed as COVID-19 death data are not available at the NUTS-3 level from official open data channels. An adjusted time-dependent SIRD model is used to predict the behavior of the epidemic; specifically, the number of susceptible, infected, deceased, recovered people and epidemiological parameters. Predictive model performance is evaluated using comparison with real data.

## 1. Introduction

The outbreak of the COVID-19 pandemic in early 2020 has caused an unprecedented effort of the scientific community to produce models that could monitor and predict the evolution of the epidemics in a reliable way, also to advise governments, in order to take prompt action which could mitigate the burden on hospitals for the treatment of infected patients, and reduce the infection mortality rate.

The first Italian COVID-19 case dates back to 20 February 2020 [1] and is reported in the city of Codogno, southern Lombardy. The epidemic spread particularly in northern Italian regions, i.e., those regions with more trading ties with China, where the pandemic had its origin. The Italian government took subsequent measures to contain the pandemic [2], ending soon with a full national lockdown on 11 March 2020, to drastically reduce the mobility of citizens and the probability of infectious contact.

The COVID-19 outbreak in Italy has not been homogeneously spreading within EU NUTS-2 regions, with many differences from province to province within the same region, and therefore it was reasonable to focus on Italian provinces (i.e., EU NUTS-3 regions), rather than on Italian regions. Differences between provinces in the same region have been noted, even in the management of the health crisis as the experience of the Lombardy region, i.e., the first Italian region hit by the pandemic, clearly shows [3,4,5].

According to the characteristics of the virus spreading, it is not suited to think of a uniform virus propagation behavior at the regional level. Even the timing of the initial stages of the infection and its dynamics seem to have been very different, even among contiguous provinces, as clusters of COVID-19 contagion have often been located in very restricted areas. One of the proofs of this lack of spread homogeneity is in the initial stage of the virus outbreak, which was located at the border between the Lombardy and Emilia-Romagna regions, namely in the Lodi province (Lombardy) and in the Piacenza province (Emilia-Romagna). The provinces of Varese in Lombardy and Ravenna in Emilia-Romagna were not comparable with the provinces of Lodi and Piacenza in terms of contagion intensity. Moreover, viral RNA swab tests had been initially conducted depending on the choices of the local health authorities, and hospital admissions in the early stages of the emergency depended on the management and absorption capacity of the local health units, resulting in many differences in practicing hospital care at a local level. Furthermore, the management of the residents of care homes was quite different locally, causing, in some cases, the development of local surges of the infection and an increase in mortality, since the illness is particularly severe for the elderly.

Compartmental deterministic models often model the spread of an epidemic [6,7], where a population of susceptible individuals evolves into other categories representing the different stages of the infection. We consider here a model consisting of 4 compartments: susceptible (S), infected (I), recovered (R), and deaths (D), which were the only compartments for which we could find available data at the NUTS-3 level in Italy.

In the case of the SARS-CoV-2 virus, it has been proven that the infection has an incubation period of about five days and that a significant percentage of the infected people are asymptomatic [8,9,10]. Thus, actually, more compartments should and have been considered, both in deterministic and stochastic models (see, e.g., [2,11,12,13,14,15,16]). Unfortunately, the data unavailability at the NUTS-3 level would cause problems in the identification of the model parameters [17]. Furthermore, we decided to keep the model as simple as possible in order to make it more accountable and, at the same time, robust to the parameter variation over time, as the infection rate depends implicitly on: (i) the citizens’ mobility and in general their social behavior; (ii) the measures of protection of the healthcare personnel and the workers who kept on doing jobs which were considered essential services to the community; (iii) the number of swab tests performed locally to detect the infected subjects in order to put them in strict quarantine. Moreover, the recovery rate and the death rate are changing in time and space because of the different burdens of the local healthcare systems, new insights into the pulmonary illness caused by the SARS-CoV-2 virus, and the evolution of its possible pharmacological and medical treatment. Finally, an adequate vaccination campaign would contribute to slowing down the pandemic. We thus implemented an adjusted the susceptible-infectious-recovered-deceased (SIRD) model, of which the parameters are evolving in time and automatically adapt to the factors which are implicitly causing changes. This variation in the parameters makes our model able to predict the short term evolution of the epidemics with good reliability, particularly in the absence of sudden oversized changes in population behavior or health policy. We leave the detection of significant change points in the parameters behavior to subsequent papers, using time series or stochastic processes techniques like the one described in [18]. For the reasons described above about the need for modeling the virus spread locally, we also implement a spatial model to allow for correlation between contiguous provinces.

Our approach belongs to the time-varying parameter SIRD models, which have been frequently used in the COVID-19 pandemic modeling literature, allowing for capturing sudden variations in the pandemic trend. For example, ref. [19] used linear combinations of basis functions to estimate pandemic parameters, using sparse identification techniques and optimizing the estimates with the Lasso technique. They applied their model to regional data; we use provincial data in an autoregressive approach, reinforced by ridge regression optimization and time series clustering techniques to reinforce the model’s training. Moreover, a STARMA spatial modeling (see Section 3.4 for details) is then used to capture the spatial correlation within the parameters. Ref. [20] integrated the classical SIRD model with artificial neural network modeling, but again using large-area data and not considering the spatial factors influencing the spread of the virus.

The rest of the paper is organized as follows. Section 2 describes the data extraction that we performed, including the extra data on deaths not available at a province-level from official authorities, together with a short discussion on issues in the data collection. It presents the model used and describes an adjusted training process that we adopted to improve the estimates. Section 3 focuses on the prediction performance of the model and an analysis of the spatial association between provinces. Section 4 discusses the paper’s main findings, while Section 5 outlines some possible future work based on the limitation of the model presented. The analyses presented in this paper were carried out using the *R* software.

## 2. Materials and Methods

### 2.1. Data on COVID-19 in Italy

Data on daily new cases of COVID-19 for each province have been made available by the Italian Presidency of the Council of Ministers-Department of Civil Protection Agency (CPA), since the very beginning of the outbreak in Italy (Official data are available at https://github.com/pcm-dpc/COVID-19, accessed on 31 May 2021). No data about deaths and recovered patients were provided at the provincial level. Therefore, we decided to integrate the official data on new cases with data on new provincial deaths derived from press conferences and reports published online by regional authorities or local newspapers. Data on deaths have been acquired using the daily press conferences and COVID-19 bulletins from regional authorities for many Italian provinces.

Regions for which we could not obtain provincial death data from official bulletins or press conferences were Lombardia and Campania. However, we obtained data on COVID-19 deaths from local newspapers for the Cremona province in Lombardy. Table 1 contains all the sources that we used to retrieve provincial COVID-19 death data.

Another time series not available from official repositories at a provincial level was the number of recovered people at time *t*. Therefore, the series was estimated using the recovery rate at the regional level, computed as the ratio of recovered people and the total number of new cases each day. The regional recovery rate at time *t* was then multiplied by the number of total cases in the province within the region:

$$R_{prov,t}=T_{prov,t}\frac{R_{reg,t}}{T_{reg,t}}$$

*R* is the total number of recovered individuals, and *T* represents the total number of cases. We estimated this number proportionally to the regional ones because the patient treatment for the illness due to COVID-19 could be considered more uniform across the provinces (with almost the same recovery rate across provinces within the region) for the number of deaths. Protocols on treatment were adopted uniformly in each province within each region.

Another data issue that researchers have faced in dealing with COVID-19 forecasting in Italy is that official data often present flaws, mainly related to delays in reporting new cases and deaths, missing data, and negative values in the series of new cases post-event recounts, and missing data. Menchetti and Noirjean [21] reported widely on the biases of these official data. Bartoszek et al. [22] highlighted that reporting statistics at a specific spatial level (national, regional, etc.) in Italy do not say much about the dynamics of the disease at lower levels. The problem of unreliable data becomes even more cogent with epidemiological models, both deterministic and stochastic, when many parameters should be estimated based on unreliable data, especially for long-range estimates, which are even more critical for an outbreak with such dramatic consequences that the whole world is experiencing. This inevitably results in less robust estimates.

**Figure 1:**
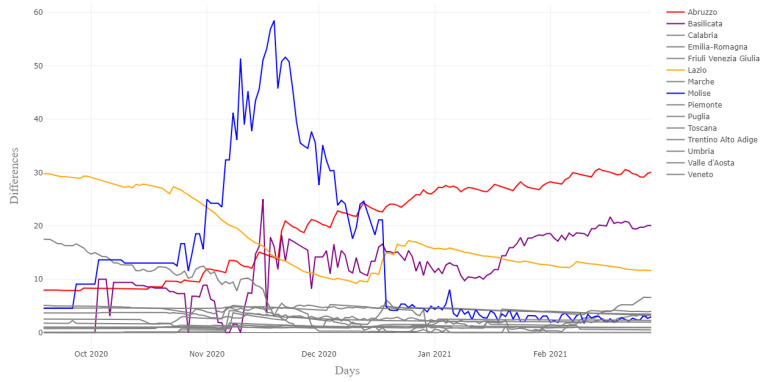
Official and retrieved data comparison.

Figure 1 reports on the differences between the sum of provincial COVID-19 cause-specific deaths and the related total regional deaths for each region, for which we have retrieved the data from regional authorities from 10 September 2020 to 28 February 2021. For a few regions, differences are often related to a delay between the publication in press conferences and the reporting in the official CPA data repository. This is an issue also experienced in other countries during the pandemic (see [23] for the number of death adjustments in the United Kingdom). Figure 1 clearly shows that for four regions out of 15, there were some discrepancies concerning the official CPA data, but these are mainly due to recounting and counting deaths “from other regions” occurring for people deceased in other regions than that of their residence, as reported by the CPA data.

However, our choice to work on death data published on regional authorities and local newspapers improved the quality of data, as the reporting was more up to date than the regional data published by CPA and avoiding the “recounting problem”, which sometimes affected the time series with peaks due to reporting delay, not present in the provincial time series. Table 2 and Table 3 better explain the two problems.

In Table 2, the numbers of daily deaths for each province in the Marche region are displayed from 1 April 2021 to 4 April 2021. At the bottom of the table, the total deaths from provincial deaths and the deaths reported from the CPA data set are displayed. This is a typical case of a region where there is no “from other regions” issue present, but the daily CPA underreporting of deaths and the huge death recounts done from time to time are of great magnitude. Note, in fact, that on April 1st, 2nd and 4th, deaths were always underreported, whereas on April 3rd, a huge recount from previous days was done. On the other hand, Table 3 is an example of a reverse issue: differences between the sum of provincial deaths and the number of regional deaths from the CPA data set are always due to the “from other regions” reporting. From these two examples, it is clear that the regional death time series is strongly affected by this recounting or delay in reporting, with unjustified peaks created from time to time, and therefore an approach taking into consideration provincial data acquired from regional bulletins and local newspapers is more appropriate, at least in the case of the Italian COVID-19 data.

### 2.2. An Adjusted Time-Dependent SIRD Model

**Figure 2:**
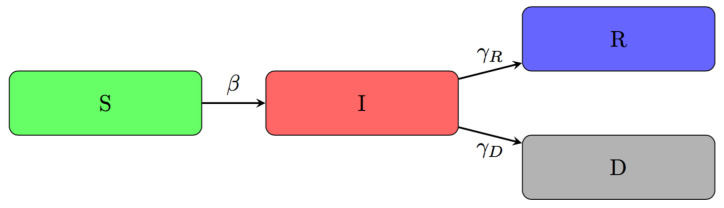
SIRD model compartments and flows.

The SIRD model is a compartmental model used in epidemiology to design the spread of a disease [6,24,25]. The model divides the population into four different groups: susceptible, infected, recovered, and deceased. This kind of design is appropriate when the disease of interest respects the following two assumptions: infected individuals can propagate the infection; recovered individuals receive longstanding immunity. The COVID-19 pandemic respects the first assumption, and some preliminary studies show that recovered individuals receive at least short-term immunity. There are other essential assumptions concerning the population (and therefore the number of susceptible people). First, its size is considered fixed (in our case, we considered Italy, which was affected by movement restrictions, sometimes with people not leaving their region). Second, individuals are identical to one another (i.e., demographic factors or different health conditions are not considered). Finally, we do not consider the effect of the vaccination campaign, as, for the period considered, it was still at the first stages in Italy. In Figure 2, a schematic of the compartments and flows forming the model is shown.

The SIRD model is based on four variables S(t), I(t), R(t), and D(t), that are respectively the number of susceptible people at the beginning of the period considered for the time series (taken from the Italian National Statistical Institute data warehouse: http://dati.istat.it/, accessed on 30 April 2020), currently infected, recovered and deaths at time *t*. The size of the population, *n*, is given by the sum of these four variables. The model’s parameters are the transmission rate, the recovery rate, and the mortality rate, respectively represented by β, $\gamma_{R}$, $\gamma_{D}$. Being rates, these parameters can also be seen respectively as the average time between effective contagious contact ($\beta^{-1}$) and the average time before removal from the infectious class (${\left(\gamma_{R}+\gamma_{D}\right)}^{-1}$).

Another important parameter that summarizes the spread of an outbreak is the basic reproduction number, $R_{0}$, which is computed as the ratio between the transmission rate and the sum of the recovery and mortality rate. Furthermore, $R_{0}$ represents the expected number of individuals directly infected by one infected individual, in a population where everyone is susceptible to infection. If $R_{0}$ is less than 1, the epidemic will eventually be controlled. If it is larger than 1, the transmission of the disease will increase in the population. The formula for $R_{0}$ is given by:

$$R_{0}=\frac{\beta }{\gamma_{R}+\gamma_{D}}$$

Building on Chen et al.’s work [26], in this paper, a time-dependent model is proposed in order to let the parameters be free to change over time. This kind of model is chosen because, in Italy and various other countries facing the virus, containment measures have been adopted and incremented over time. In particular, a national lockdown was introduced in Italy on 11 March 2020, and lasted until 4 May 2020. Other pandemic containment measures were taken later on. By allowing the parameters, especially the effective transmission rate, to vary over time, control measures can be somewhat included in the model. On the other hand, recovery and mortality rate are likely to depend on the pressure under which hospitals and, in particular, intensive care units are in, which increases sharply at the beginning of a pandemic (i.e., when a high mortality rate is reported) and then relaxes after the health system capacity is enhanced.

The differential equations governing the standard deterministic SIRD model are the following:

$$\begin{array}{ccc} \frac{dS}{dt} & = & -\frac{\beta (t)S(t)I(t)}{n}\\ \frac{dI}{dt} & = & \left(\frac{\beta (t)S(t)}{n}-\gamma_{R}\left(t\right)-\gamma_{D}\left(t\right)\right)I\left(t\right)\\ \frac{dR}{dt} & = & \gamma_{R}\left(t\right)I\left(t\right)\\ \frac{dD}{dt} & = & \gamma_{D}\left(t\right)I\left(t\right) \end{array}$$

subject to the constraint S(t)+I(t)+R(t)+D(t)=n, since we are neglecting the effects of new births and of people dying for causes not related to COVID-19. Note that, because of this constraint, one of the previous equations in the SIRD model can be derived from the other ones, and can be omitted.

We consider the day as a unit of time, and we transform the previous system of ordinary differential equations into a discrete-time difference system of equations, using Δt=1 and applying a forward finite differences scheme, which results in the following:

(1) $$\begin{array}{ccc} S(t+1)-S(t) & = & -\frac{\beta (t)S(t)I(t)}{n}\\ I(t+1)-I(t) & = & \left(\frac{\beta (t)S(t)}{n}-\gamma_{R}\left(t\right)-\gamma_{D}\left(t\right)\right)I\left(t\right)\\ R(t+1)-R(t) & = & \gamma_{R}\left(t\right)I\left(t\right)\\ D(t+1)-D(t) & = & \gamma_{D}\left(t\right)I\left(t\right) \end{array}$$

From the records of the four variables of interest in a specific province, the evolution of each parameter can be retrieved using the equations above, as follows:

$$\begin{array}{ccc} \beta (t) & = & \frac{n(S(t+1)-S(t))}{S(t)I(t)}\\ \gamma_{R}\left(t\right) & = & \frac{R(t+1)-R(t)}{I(t)}\\ \gamma_{D}\left(t\right) & = & \frac{D(t+1)-D(t)}{I(t)} \end{array}$$

The observed time series of S,I,R,D is then used to estimate the daily values of transmission rate, recovery rate, and mortality rate and predict future values. Because of the stochasticity of the time series, the daily estimate of the three driving parameters is itself stochastic, and we need then to smooth or filter out the noise of the estimates in order to obtain robust predictions.

We do not assume any specific underlying model for the noise, and, in order to make our predictions more robust, we decided to apply a finite impulse response (FIR) filter. The following equations describe the regression model used in the FIR filter for each parameter and the cost function to be minimized in order to find the optimal coefficients:

$$\begin{array}{c} \hat{\beta }\left(t\right)=c_{0}+\sum_{j=1}^{J}c_{j}\beta \left(t-j\right)+c_{s}s_{t-14}\\ \hat{\gamma_{R}}\left(t\right)=c_{0}+\sum_{j=1}^{J}c_{j}\gamma_{R}\left(t-j\right)\\ \hat{\gamma_{D}}\left(t\right)=c_{0}+\sum_{j=1}^{J}c_{j}\gamma_{D}\left(t-j\right)\\ min_{c}\left(\sum_{t=J}^{T}{\left(Y\left(t\right)-\hat{Y}\left(t\right)\right)}^{2}-\lambda ∥c∥\right) \end{array}$$

where $c_{0}$ and $c_{j}$ are the usual intercept and regression coefficient parameters. J was set to 14, since the estimated period change in the pandemic is likely to become visible in the data after 14 days, as this is also the quarantine time used in Italy.

The FIR filter requires a single hyper-parameter (*J* in the above formula) which represents the maximum number of lag days to include in the regression. The cost function consists of a regularized least-squares method in which the penalty (λ) is applied to the sum of squares of the regression coefficients (Ridge regression regularization, based on a $\ell_{2}$ norm [27]). Different penalty functions λ have been used in the ridge regression to estimate each parameter, i.e., transmission, recovery, and death rates. The value of λ for each parameter has been obtained using cross-validation. Therefore, the resulting overall model is such that its parameters are time-dependent, and the lags of these are modeled via loss functions, whereby parameters at a time are regressed on previous lagged parameters.

The SIRD models suffer from some drawbacks in periods of fast pandemic spread or contraction (they tend to overestimate when there is a rapid increase of the infections and to underestimate when there is a sudden decrease, see [28,29]). In order to consider the effect of the social distancing policy adopted in Italy, the transmission parameters are multiplied day to day by a parameter $\psi_{t}=1-s_{t}$, where $s_{t}$ is the national COVID-19 stringency index in that period as computed according to [30]. We present model evaluation results with and without considering the stringency index in Section 3.1.

Once the model has been trained using historical data, future predictions can be made on the parameters and, therefore, estimates for the evolution of *S*, *I*, *R*, and *D* can be computed using the SIRD model equations.

### 2.3. Adjusted Training Process

Our model aims to make predictions about the evolution of the COVID-19 outbreak in Italy at the local level, particularly using historical data on each province. The model presented here differs from [26], in that the hyperparameters are not considered fixed, but optimized using multiple approaches, and it is composed of three different autoregressions based each on a SIRD model’s parameter. Each of the regressions requires the choice of the penalty value for the regularization process (λ). In our approach, the regularization parameters were free to vary (within a range from $-10^{-5}$ to $10^{5}$ with step 0.1 for the powers), and cross-validation was employed to find their optimal values [31].

**Figure 3:**
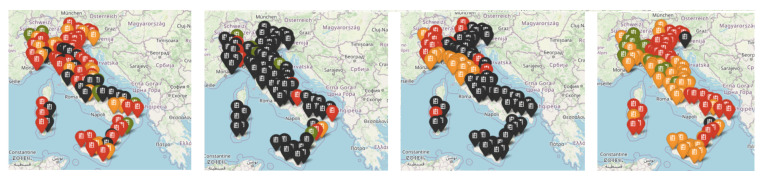
Comparison of $R_{0}$ index values on 10 September 2020, 10 October, 10 November, 10 December, for provinces where the number of deaths is available. Green pins are for $0\leq R_{0}<0.5$, orange pins for $0.5\leq R_{0}<1$, red pins for $1\leq R_{0}<2$ and black pins for $R_{0}\geq 2$.

However, the deterministic model described above might struggle to produce reliable estimates in contexts where the number of cases or deaths is meager. There is considerable fluctuation or inconsistency in the data, as happens in some provinces where the outbreak is not so intense (see Figure 3, where the heterogeneity of the outbreak is clearly shown at a provincial level, at least in the first-medium stages of its evolution). In contrast, it seems to give more robust results when data are aggregated at a higher level, as in the entire country’s time series. This happens because the model is based on smoothing the sequential values of the variables that become less precise as the numbers decrease. Therefore, an aggregation approach was used to train the three models, based on the assumption that provinces which ’behaved’ similarly in recent history are more likely to behave similarly in the future.

For a given province and a given forecast origin, three sets containing the most similar provinces concerning the different parameters β, $\gamma_{R}$, and $\gamma_{D}$ were retrieved. The distance between the two series was computed using dynamic time warping (DTW) with Itakura constraint to allow for some small temporal shifts between the series [32]. The similarity was considered only in the last 30 days before the origin, assuming that only the most recent past was of interest. The number of provinces to select for each set was chosen using cross-validation: the first decile, corresponding to 9 provinces, resulted in the minimum prediction error. Such a choice is also reasonable in terms of the problem that we are trying to solve, since it appears to be a good compromise between the overfitting and extreme generalization of the series.

**Figure 4:**
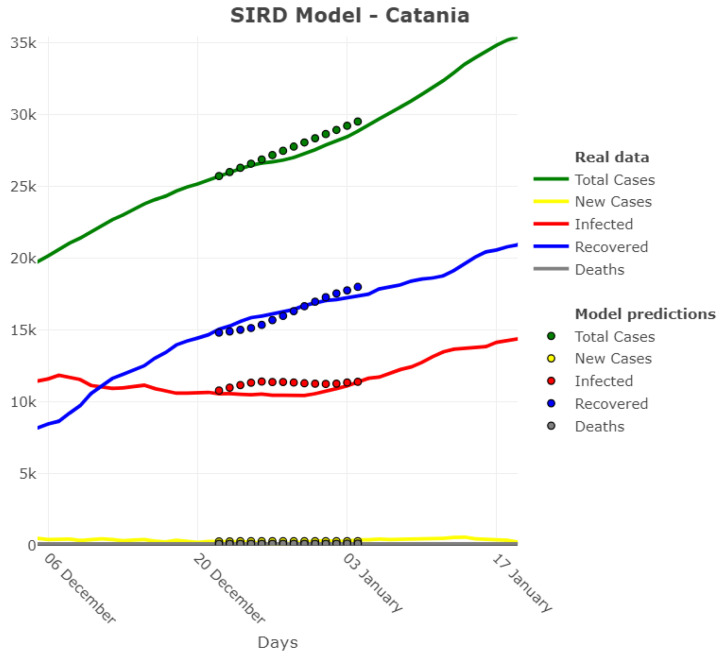
Adjusted SIRD model for Catania province—Forecast origin: 20 December 2020.

In Figure 4, an example on applying this model to parameter *I*, *R* and *D* for the Catania province is shown.

The predicted values for these parameters are derived from the predicted hyperparameters β, $\gamma_{R}$, and $\gamma_{D}$. Together with these estimates, the estimates for the total cases and the new daily cases are also obtained algebraically and displayed in the figures.

Predictions for the hyperparameters and other provinces, together with other model settings like the number of lags to be chosen for prediction, can be seen on a dashboard developed for this model, available at https://ceeds.unimi.it/covid-19-in-italy/ (accessed on 31 May 2021, see also [33] for a detailed description of this dashboard).

### 2.4. Bootstrap Prediction Intervals

Because of the numerous issues causing inconsistency in the reported daily data, it is necessary to accompany the point estimates with reasonable confidence intervals. Since the model starts with predicting the disease parameters, the intervals are also computed first on the parameters and later derived for I, R, and D.

In order to build prediction intervals for the model parameters, a block bootstrap algorithm using a stationary version of the time series with blocks of 30 observations was used [34]. Zero lower bound was imposed for all the parameters to avoid results that contradict the compartmental logic of the SIRD model. The same procedure could be applied to the three regressions of the model, respectively used for β, $\gamma_{R}$, and $\gamma_{D}$. However, each of the model’s variables depends on the whole set of parameters, so that the obtained error range for a parameter must be combined with the other two parameters’ intervals. Thus, the intervals for the variables are subject to the uncertainty of three different parameters and can be composed in different ways. Combining lower and upper bounds of the parameters can be misleading, since the variables of the SIRD model develop in different directions and, mainly, each variable depends on the past value of I(t), which in turn depends on S(t−1), R(t−1), and D(t−1). Nevertheless, combinations of interest can be used to describe the epidemic development in particular scenarios. The method proposed here is to use the prediction interval for the parameter of interest and use point estimates for the other two parameters. Thus, the effects of the variability of the parameter can be easily displayed on each variable. Accordingly, when the parameter of interest is β, the following equations are used to compute the prediction intervals for the variables *S*, *R*, *D* and *I*, following Equation (1):

$$\begin{array}{ccc} S{\left(t+1\right)}_{low/up} & = & S{\left(t\right)}_{low/up}\left(1-\frac{\beta {\left(t\right)}_{up/low}I{\left(t\right)}_{up/low}}{n}\right)\\ R{\left(t+1\right)}_{low/up} & = & R{\left(t\right)}_{low/up}+\gamma_{D}\left(t\right)I{\left(t\right)}_{low/up}\\ D{\left(t+1\right)}_{low/up} & = & D{\left(t\right)}_{low/up}+\gamma_{R}\left(t\right)I{\left(t\right)}_{low/up}\\ I{\left(t+1\right)}_{low/up} & = & n-S{\left(t+1\right)}_{up/low}-R{\left(t+1\right)}_{low/up}-D{\left(t+1\right)}_{low/up} \end{array}$$

where low and up stand respectively for lower and upper bounds.

**Figure 5:**
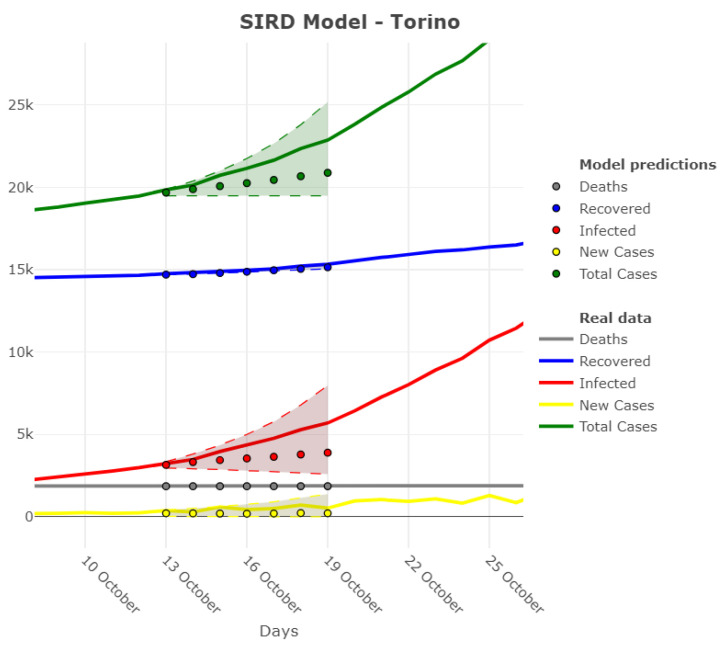
Variables’ prediction intervals based on β interval—Torino province starting from 12 October 2020. The shaded region represents the 90% confidence bands, while the dots represent the point predictions. Real values are always within the confidence bands.

Bootstrap intervals for each variable are shown in Figure 5 for the province of Torino. Note that real values in the prediction windows are always within the confidence bands.

### 2.5. Model Evaluation

The model performance was evaluated using two commonly employed accuracy metrics. The mean absolute percentage error (MAPE) is a popular error measure used to assess the reliability of model prediction and is widely used in medical research (see, for example, [35,36] or [37]). In this application, the MAPE was employed to estimate the average absolute error of the model on the different forecasting horizons:

$$\begin{array}{ccc} MAPE_{h} & = & \frac{100}{PD}\sum_{p=1}^{P}\sum_{d=1}^{D}\left|\frac{Y_{hpd}-{\hat{Y}}_{hpd}}{Y_{hpd}}\right| \end{array}$$

where *h* is the forecast horizon, *P* is the number of provinces in the sample, *D* is the number of days in the sample, ${\hat{Y}}_{h}$ is the resulting prediction, and $Y_{h}$ is the real value for the variable.

The mean percentage error is also commonly employed in the literature ([37]), and it helps in the understanding of the distribution of the errors over the different units under investigation. Thus, it was explicitly computed for every combination of province and horizon.

$$\begin{array}{ccc} MPE_{hp} & = & \frac{100}{D}\sum_{d=1}^{D}\frac{Y_{hpd}-{\hat{Y}}_{hpd}}{Y_{hpd}} \end{array}$$

### 2.6. Model Extensions

In the previous sections, each province had its specific model trained using a cluster formed by the most similar provinces in terms of the parameters. The distance between provinces was used assuming that provinces with similar behavior in the near past will continue to have that behavior in the future, despite the geographical distance. The spatial structure was not considered, as it was assumed that neighboring provinces did not affect one another.

Indeed, neighboring provinces are likely to influence one another in many ways. Movements between bordering provinces are more likely to happen than between non-bordering ones. For example, commuters (i.e., potential spreaders) are more likely to work or study in a neighboring province, thus increasing the probability of exporting (or importing) the virus in nearby territories. Moreover, during the period of emergency, residents of different provinces shared medical resources, such as hospitals, health care workforce, equipment, and test capacity, thus affecting several different parameters regarding the spread of the epidemic, such as the reported rate of transmission and the reported number of COVID-19-related deaths.

Given these considerations, a spatial model is also implemented. Its results are presented in Section 3 as a possible alternative to the original strategy for spatial effects. The model performance is not considered in detail, as the focus of this article is the methodology presented in Section 2.2 and Section 2.3, while the spatial model only represents one of the possible extensions to the original model that could be developed when less restrictive assumptions are made. Other potential models may consider the hierarchical structure of the national, regional, and provincial levels, or could drop the assumption of independence between the three parameters $\beta_{t},\gamma_{R}$, and $\gamma_{D}$.

## 3. Results

### 3.1. Model Accuracy Evaluation: Day-By-Day Forecasting Evaluation

The model accuracy was evaluated on the training approach presented above. In order to calculate the MAPE for different values of *h*, we applied the model to all the provinces within a sample period, lasting from 2 October 2020 to 25 February 2021 (D=146), corresponding to the second wave of the epidemic in Italy. For each day of the period, the model predicts the values of the various pandemic variables, using a time horizon from 1 to 14 days ahead in the future.

The regularization parameter λ is free to vary across provinces and days. Model predictions are then compared with the real values. Results are shown in Table 4, where we present MAPE values, both with and without considering the daily national stringency index presented in Section 2.2.

MAPE values are relatively low for all of the variables in the short term. As is usual in time series forecasting, the error increases as the horizon for the prediction becomes larger: nevertheless, our model performance remains acceptable, even in the longer term. The estimate whose MAPE is the highest is for *I*, whereas the MAPE for the deaths (‘MAPE D’) is always the lowest, whatever the horizon. Finally, the MAPE for the recovered (‘MAPE R’) is always in the middle. Note that MAPE D has a MAPE under 10% until day 12. MAPE R is under 10% until day 7 and MAPE I until day 3.

For horizon days 10–14, MAPE values could appear relatively high. This is because the time window that we considered included the second COVID-19 wave (October 2020–December 2020), which has been the worst since the beginning of the pandemic. When infection dynamics change and the numbers become more considerable, SIRD models, in general, tend to lead to an increase in prediction errors. Our results on medium-term forecasts are in line with other results in the recent COVID-19 forecast literature, like in [38,39,40].

MAPE values computed considering the national daily stringency index are generally larger than those computed without it. This could be since we are taking into account the national stringency index, whereas we should have considered the provincial, which is not currently available. However, the maximum relative change in MAPE between those with and without stringency index (considering the latter as reference) is around 5%. For the number of deaths *D*, until the 4th horizon day, the MAPE with the stringency index is lower or equal than the MAPE without stringency index. For this parameter, the relative change is always under 1%.

As a sensitivity analysis, we have also run the model without the four regions having more data discrepancies in terms of comparison between retrieved and official data highlighted in Figure 1. Table 5 shows the MAPE D relative to the subset of data without these four regions for provincial aggregating, revealing only tiny differences for column ‘MAPE D’ in Table 4. However, for each horizon, there is a slight improvement for the MAPE values in Table 4, hinting at an even better performance with high-quality data.

Therefore, this model training can be considered optimal, and the analysis of its forecasting reliability is further developed.

### 3.2. Model Accuracy Evaluation: Error Distribution across Provinces

The distribution of the mean percentage error (MPE) [41] was also analysed for each province to understand more about the prediction reliability of our model, given different time horizons.

**Figure 6:**
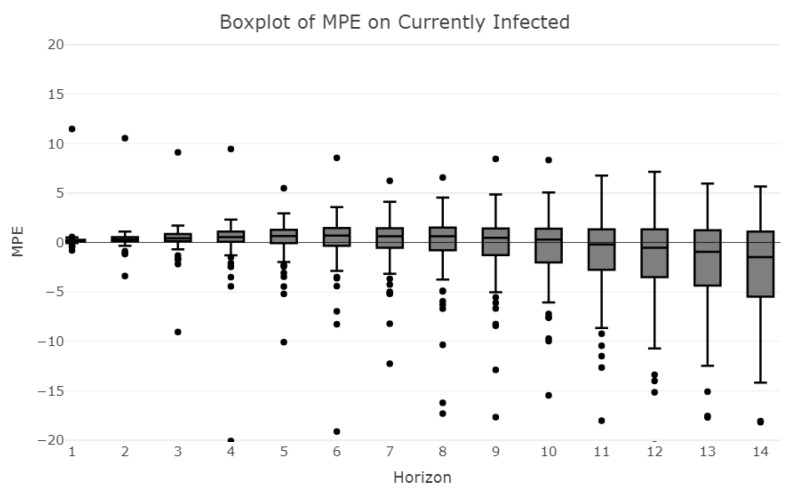
Boxplots of MPE on currently infected.

The mean is computed over the period considered, so that each value represents the average error that the model makes in the specific context of a province; Figure 6 shows box plots of the distributions of provincial forecasts, given the forecast horizon.

For a time horizon lower than five days, 75% of the provinces are accurately predicted with percentage errors lower than 10%. A time horizon equal to 6 days still shows an acceptable level of error with almost all provinces below the 20% threshold. Even when the time horizon covers 13 days, half of the provinces show errors below 25%, meaning that although the overall accuracy decreases, the model still performs pretty well in some provinces.

### 3.3. Clustering the Provinces by One-Week Errors on I

**Figure 7:**
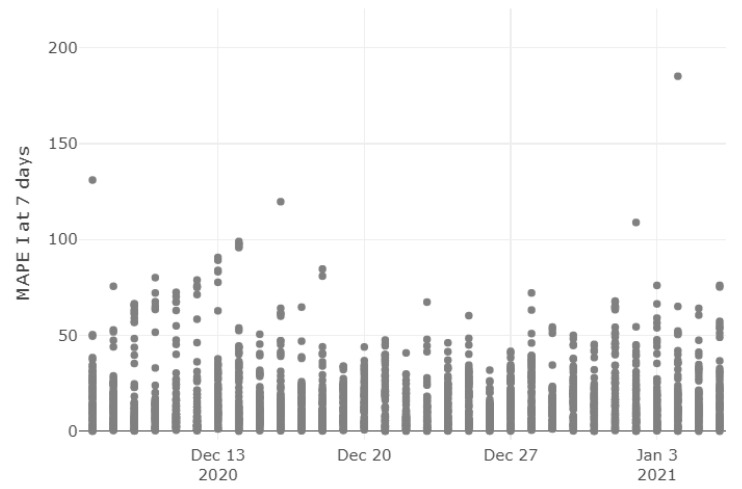
MAPE on weekly predictions over time. The dates on the horizontal axis represent the time window limit on which the training was done. The vertical axis displays the absolute error between real and predicted on the seventh day after that date for each province.

Based on the error distribution analysis conclusions, weekly predictions are considered the optimal context of an application for our model. In this section, the MAPE on currently infected people is analyzed using a one-week horizon. Figure 7 shows how the one-week prediction error varies when we start to forecast those currently infected on different days. We can see that there are no alarming shifts in the model accuracy and that a large majority of the provinces are even below the 20% threshold. Although this happens in the sample period of our choice, the model accuracy had likely improved since the beginning of the epidemic, when most provinces reported very low numbers of cases, making the predictions harder.

### 3.4. Spatial Model

The presence of a spatial autocorrelation structure across the provinces appears evident in the analysis of the Moran’s index [42], which is a measure of association between geographical units and their neighbors on the number of infected individuals. In the computation of the statistic, first-order neighbors, (i.e., adjacent provinces) were considered to design a spatial weight matrix with each row and column representing a province: if the provinces do not share a border, then a weight of 0 is assigned; the weight for the neighbor provinces is assigned so that the row-wise sum is equal to 1. Moran’s index (Formula 2) is computed for each day using *I* as the variable of interest: *p* stands for the number of provinces; $I_{it}$ is the reported number of active cases at time *t* in province *i*:

(2) $$\begin{array}{ccc} I_{t} & = & \frac{p}{\sum_{i=1}^{p}\sum_{j=1}^{p}w_{ij}}\frac{\sum_{i=1}^{p}\sum_{j=1}^{p}w_{ij}\left(I_{it}-{\bar{I}}_{t}\right)\left(I_{jt}-{\bar{I}}_{t}\right)}{\sum_{i=1}^{p}\left(I_{it}-{\bar{I}}_{t}\right)} \end{array}$$

**Figure 8:**
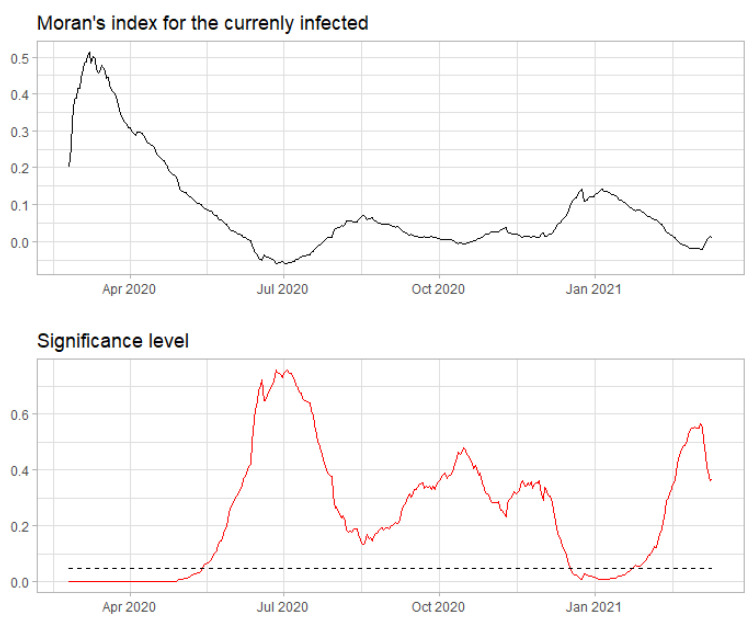
Moran’s index computed on *I* using a row-standardized spatial weight matrix. The lower panel gives the significance levels corresponding to each day of the time window considered, and the horizontal dotted line is the 0.05 *p*-value level.

The spatial association is significantly different from 0, particularly in periods of severe outbreaks (Figure 8). In the first days of the epidemic, the spatial autocorrelation shows a substantial significance, and its inclusion in the model might improve the forecasting process. In the period in which the pandemic situation is improving, i.e., from late April–early May 2020, the association between provinces and their neighbors tends to decrease, thus suggesting that the spread of the virus might have become more homogeneous within the national territory. The steep long-lasting decrease in Moran’s index value from late April–early May 2020 might be due to the ease of restrictions and the fast decrease in the number of new cases reported throughout Italy in that period. It was expected that the spatial autocorrelation grew again in the autumn, as the long-distance mobility imputable to vacation traffic decreases and local outbreaks become more likely than extended ones.

The spatial association between bordering provinces can be considered in the model by including spatial lags and temporal ones. Such models, including both temporal and spatial lags, are space-time autoregressive moving average (STARMA) models ([43,44]). STARMA models are helpful when there is dependence between contiguous regions, and therefore are effective to model pandemic situations—like the one of COVID-19 in Italy—thus in line with our approach of modeling the virus spread more locally, and have been adopted in many spatial contexts [45]. Moreover, considering the current situation in other provinces, predicting the epidemic evolution in a particular region can be an alternative to the aggregation training approach presented in the article. Including time lags for all the neighbor provinces is costly in terms of the number of coefficients to estimate, so that the regularization technique (Ridge) used in the first model becomes increasingly valuable for the spatial model, to perform the shrinkage of the coefficients and enhance the generalization ability of the model, needed to make robust predictions.

The STARMA model, including both temporal and spatial lag, can be summarized in the following equation that models the vector of parameters $Y_{p,t}$ containing $\beta_{t},\gamma_{R},\gamma_{D}$:

(3) $$\begin{array}{c} Y_{p,t}=\sum_{j=1}^{J}\phi_{p,j}Y_{p,t-j}+\sum_{i\in \mathrm{neighs}(p)}\sum_{j=1}^{J}\phi_{i,j}Y_{i,t-j}+\varepsilon_{t} \end{array}$$

where neighs(p) returns the neighbour provinces of *p*, *J* is the number of lags to use that was set to 14, $\phi_{i,j}$ represents the coefficient for each combination of lags i,j. The regularization is performed independently for each of the three parameters using cross-validation to find an optimal value for λ.

**Figure 9:**
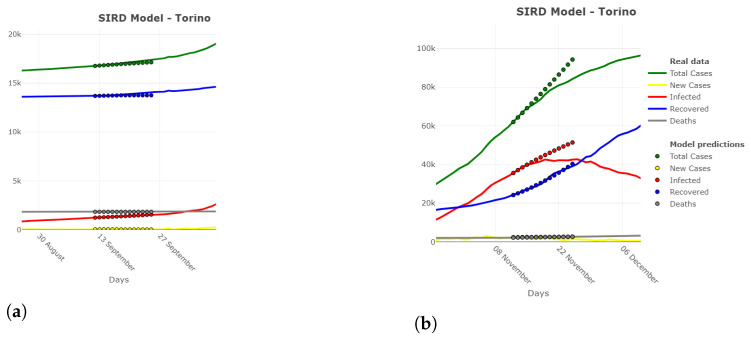
SIRD model predictions for Turin province using the STARMA model based on data up to September 10th and November 30th, 2020. (**a**) September 10th; (**b**) November 30th.

The specified model can be trained for all of the parameters, and it can be used to predict their future values. Once the forecasting is done for β(t), $\gamma_{R}\left(t\right)$, and $\gamma_{D}\left(t\right)$, the SIRD variables can be computed in the usual way using Equation (1) and Equation (3). An example of the outcomes from the implementation of this autoregressive space-time model can be seen in Figure 9 for the province of Turin.

Like in the case of the MAPE values, also, in this case, forecasts seem satisfactory in the short term, whereas in the medium–long term, the STARMA model does not seem to anticipate changes in the slope of the number of infected people series (and consequently of the total cases which are derived from them), whereas for new cases, deaths and recovered people, the model fits well. However, in November 2020 (which is the case displayed in Figure 9b), many measures to contain the spread of the virus have been adopted by the Italian government, which we did not consider in this application. However, when considering a mild increase of the virus spread (Figure 9a), the STARMA model seems to perform better.

## 4. Discussion

The COVID-19 outbreak has made it necessary to impose strong containment measures. The spread of the disease in Italy involved the whole Italian territory heterogeneously, see [12]. The total lockdown of industrial and other productive activities (from 22th March) and other restriction measures adopted later on were legitimized by few municipalities with an ever-increasing number of cases (and deaths), by the inability to stop the contagion by the emergence of new outbreaks. It is also true that the lockdown also stopped municipalities barely touched from the COVID-19 spread.

The analysis presented in this paper can be applied at the NUTS-3 region level in Italy to predict the future development of the epidemic in the specific provincial context. This choice is aimed at tackling the heterogenei0y of the COVID-19 pandemic on the territory. Furthermore, as expected in an emergency, some information may be temporarily incomplete. In particular, during the first virus outbreak, especially in provinces where there have been a high number of infections, patients, and hospitalized patients, there was the possibility of a delay of a few days between the time of the swab for diagnosis and data reported on the dedicated platform (https://www.epicentro.iss.it/en/coronavirus/sars-cov-2-integrated-surveillance, accessed on 31 May 2021).

The DTW training approach presented in this paper has been proven to be accurate, and it is therefore recommended.

The main issue found during our model building was the lack of detailed and consistent data about the epidemic at the provincial level. However, the procedure adopted by the Italian central government and the regional authorities was standardized in order to obtain similar aggregated data across regions. A full trustfulness of these aggregated data is put in doubt by the fact that some of them (deaths, recovered and a number of tests) are not publicly available at a provincial level in the official daily releases by the Italian Civil Protection Agency, and therefore, as in our case, they must be estimated or found in some ways. If more variables had been available, the model could have been extended to include other compartments, such as the hospitalized cases or the number of tested individuals, or, again, the possibility to consider a percentage of recovered people as susceptible, or the asymptomatic cases, or the underreporting of deaths. Indeed, we choose to implement the simplest (with the lowest number of parameters) model, which allows us to overlook strong and unlikely assumptions. Indeed, it is known that compartmental models are not identifiable when the parameters (more than one) cannot be directly estimated by the observed evolving compartments (see, e.g., [17]). As a consequence, one might not detect hidden variables. However, the observed positive cases likely present a severe clinical situation, and, in force of this, they more likely present the highest number of neutralizing antibodies, see [46]. The recovered individuals ’ data were also not available and, therefore, an estimate using regional data was necessary. Finally, the state of emergency in which the data was collected is likely to affect its quality and consistency. However, for the kind of modeling that we chose, relying on aggregate official data, retrieved directly or indirectly, was the only way to have reliable results. Nevertheless, the model seems to perform relatively well in the short-term horizon, and with the deaths data, we were able to acquire from regional authorities and local newspaper websites.

## 5. Conclusions

The analysis presented in this paper can be helpful to gain a general understanding of the epidemic development in the short term at the local level. Notably, it could be implemented to monitor and signal local areas at greater risk.

Along with the forecasting of the SIRD variables, the model offers other insights into the epidemic development. The computation of the parameters provides informative time series evolution of the disease in each specific context. Future work will be about considering an extended stochastic version of the adjusted SIRD model presented in this paper (for example, building on the work by Zimmer et al. [47]), the Bayesian framework, multiple-source models as in [48] and the vaccination campaigns.

## Figures and Tables

**Table 1:** Main data sources for provincial COVID-19 deaths.

Region	Main Source
Valle d’Aosta	https://github.com/pcm-dpc/COVID-19 (accessed on 31 May 2021)
Pedmont	https://www.regione.piemonte.it (accessed on 31 May 2021)
Lombardy	https://www.laprovinciacr.it (only for Cremona province) (accessed on 31 May 2021)
Veneto	https://www.ilgiornaledivicenza.it (accessed on 31 May 2021)
Friuli-Venezia-Giulia	https://www.regione.fvg.it (accessed on 31 May 2021)
Trentino-Alto-Adige	https://github.com/pcm-dpc/COVID-19 (accessed on 31 May 2021)
Emilia-Romagna	https://www.regione.emilia-romagna.it (accessed on 31 May 2021)
Liguria	https://www.regione.liguria.it (accessed on 31 May 2021)
Tuscany	https://www.toscana-notizie.it (accessed on 31 May 2021)
Marche	http://www.regione.marche.it (accessed on 31 May 2021)
Umbria	https://public.tableau.com/ (accessed on 31 May 2021)
	https://regione.umbria.it (accessed on 31 May 2021)
Lazio	https://www.romatoday.it (accessed on 31 May 2021)
	https://www.facebook.com/SaluteLazio (accessed on 31 May 2021)
Abruzzo	https://www.regione.abruzzo.it (accessed on 31 May 2021)
Molise	https://www.molisenews24.it/regione (accessed on 31 May 2021)
Puglia	http://www.regione.puglia.it (accessed on 31 May 2021)
Basilicata	https://www.regione.basilicata.it (accessed on 31 May 2021)
Calabria	https://www.regione.calabria.it (accessed on 31 May 2021)
Sicily	https://www.regione.sicilia.it (accessed on 31 May 2021)
Sardinia	https://www.regione.sardegna.it (accessed on 31 May 2021)

**Table 2:** Differences between CPA death counts and regional bulletins and local newspapers death counts—Marche region, period 1 April 2020–4 April 2020.

Province	1 April 2020	2 April 2020	3 April 2020	4 April 2020
Ancona	7	10	6	7
Pesaro-Urbino	10	20	7	14
Fermo	0	0	2	3
Ascoli Piceno	0	1	1	0
Macerata	9	0	1	1
“From other regions”	0	0	0	0
Marche (from provincial deaths)	26	31	17	25
Marche (from CPA reporting)	25	26	54	17

**Table 3:** Differences between CPA death counts and regional bulletins and local newspapers death counts—Emilia-Romagna region, period 1 April 2020–4 April 2020.

Province	1 April 2020	2 April 2020	3 April 2020	4 April 2020
Piacenza	25	19	18	12
Parma	24	11	9	25
Reggio-Emilia	9	9	14	15
Modena	10	18	9	6
Bologna	3	7	31	10
Ferrara	1	3	3	1
Ravenna	4	1	0	2
Forlì.Cesena	4	3	2	1
Rimini	5	4	4	2
“From other regions”	3	4	1	1
Emilia-Romagna (from provincial deaths)	85	75	90	74
Emilia-Romagna (from CPA reporting)	88	79	91	75

**Table 4:** MAPE values-DTW aggregation training with and without stringency index.

Horizon Days	I	I	R	R	D	D
	without s.i.	with s.i.	without s.i.	with s.i.	without s.i.	with s.i.
1	2.78	2.79	1.46	1.46	1.08	1.07
2	5.19	5.24	2.69	2.70	1.96	1.95
3	7.79	7.88	4.00	4.01	2.82	2.81
4	10.48	10.64	5.39	5.42	3.67	3.67
5	13.18	13.48	6.88	6.94	4.52	4.53
6	15.73	16.30	8.54	8.61	5.33	5.34
7	17.55	18.48	9.90	10.07	6.00	6.03
8	19.64	20.64	11.00	11.37	6.69	6.73
9	21.97	22.98	11.90	12.41	7.37	7.43
10	24.35	25.39	12.80	13.39	8.04	8.10
11	26.72	27.85	13.64	14.27	8.71	8.77
12	29.09	30.36	14.40	15.05	9.36	9.45
13	31.33	32.80	15.12	15.78	10.02	10.13
14	33.55	35.37	15.88	16.53	10.64	10.77

**Table 5:** MAPE D-Aggregation training without Abruzzo, Basilicata, Lazio and Molise without stringency index.

Horizon Days	MAPE D
1	0.987
2	1.785
3	2.560
4	3.346
5	4.115
6	4.846
7	5.458
8	6.073
9	6.706
10	7.342
11	7.986
12	8.609
13	9.245
14	9.881

## Data Availability

The dataset used in this paper can be retrieved at https://github.com/CEEDS-DEMM/COVID-Pro-Dataset (accessed on 31 May 2021). Source code will be made available with open access.

## Abbreviations

The following abbreviations are used in this manuscript:

DTW	Dynamic Time Warping
SIRD	Susceptible-Infectious-Recovered-Deceased
CPA	Civil Protection Agency
FIR	Finite Impulse Response
MAPE	Mean Absolute Percentage Error
MPE	Mean Percentage Error
STARMA	Space Time AutoRegressive Moving Average
